# Acute Chest Pain and ST-Segment Changes in a Patient After Permanent His-Bundle Pacing Pacemaker Implantation

**DOI:** 10.1016/j.jaccas.2024.103203

**Published:** 2025-02-12

**Authors:** Satoshi Yanagisawa, Yasuya Inden, Yukiomi Tsuji, Rei Shibata, Toyoaki Murohara

**Affiliations:** a Department of Cardiology, Nagoya University Graduate School of Medicine, Nagoya, Japan; b Department of Advanced Cardiovascular Therapeutics, Nagoya University Graduate School of Medicine, Nagoya, Japan

**Keywords:** acute myocardial infarction, conduction system pacing, electrocardiography, His-bundle pacing, nonselective His-bundle pacing, ST-segment elevation

## Abstract

Diagnosis of ST-segment changes is challenging in patients with right ventricular pacing rhythm. Herein, we present a patient with a history of permanent His-bundle pacemaker implantation who developed chest pain and ST-segment changes on electrocardiography. An immediate diagnosis of acute myocardial infarction on the basis of the electrocardiographic abnormality and prompt management of coronary intervention resulted in a short door-to-balloon time of 80 minutes, even on a holiday morning, and a stable clinical course thereafter. This scenario underscores the potential benefit of electrocardiographic diagnosis in physiological pacing using a native conduction system, associating prompt treatment with a favorable prognosis.

## Graphical Abstract

**Figure:**
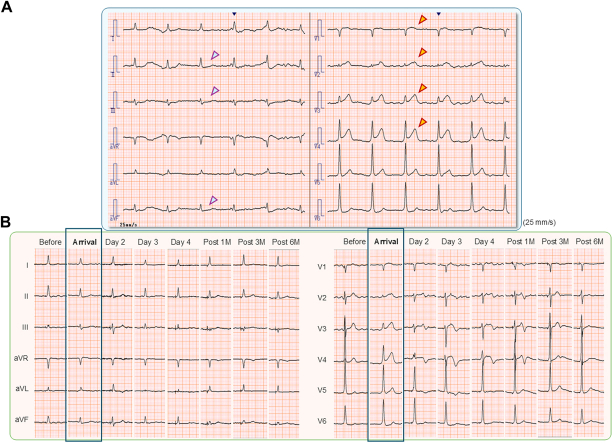

A 78-year-old man visited our emergency department on a Sunday morning with persistent chest pain for 2 hours. The patient had undergone permanent pacemaker implantation for complete atrioventricular block and atrial fibrillation bradycardia 15 months previously, with successful achievement of His-bundle pacing (HBP) (SelectSecure model 3830, Medtronic) with a continuous, stable His-bundle capture presented with nonselective HBP. In the initial examination, surface 12-lead electrocardiography (ECG) showed marked ST-segment elevation in leads V_1_ to V_4_ and depression in the inferior leads (Figure 1A). A resident fellow called the cardiology staff immediately after seeing the electrocardiogram with suspicion of acute myocardial infarction, and emergency coronary angiography was planned. The patient was treated with stent implantation following thrombus aspiration for a proximal lesion of the left anterior descending coronary artery with 99% stenosis, resulting in full coronary flow recovery and complete resolution of chest pain (Supplemental Figure 1). The patient had a stable clinical course, with a 1-day stay in the intensive care unit and discharge after 14 days of cardiac rehabilitation. The peak creatinine kinase level was 2,512 U/mL, with no accompanying complications such as heart failure or cardiac tamponade. The door-to-balloon time was excellent (80 minutes) despite the holiday morning operation. Six-month follow-up ECG demonstrated recovery of ST-T change with almost normal features (Figure 1B) and stable capture of HBP without an increased pacing threshold (Supplemental Figure 2).

**Take-home messages**

• HBP has the potential benefit of electrocardiographic diagnosis in physiological pacing using a native conduction system.
• Marked ST-T changes on ECG in nonselective HBP with concordant electrical dynamics between the QRS complex and T wave can help diagnose ischemic abnormalities, resulting in prompt treatment and a favorable prognosis in a patient with acute myocardial infarction.

**Figure 1:**
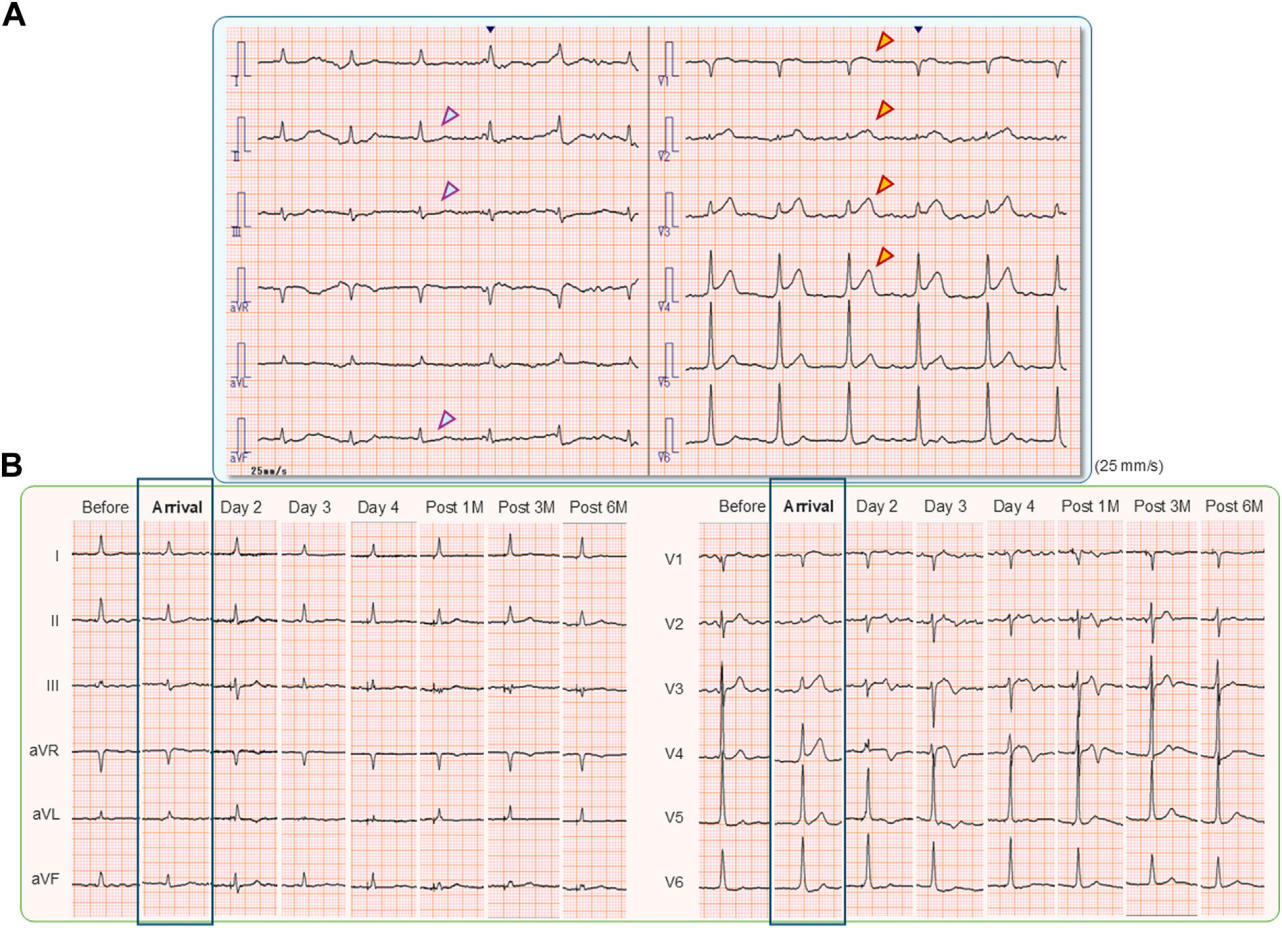
Surface 12-Lead Electrocardiography During the Clinical Course (A) On arrival with acute chest pain. Marked ST-segment elevation in leads V_1_ to V_4_ (red arrows) and significant ST-segment depression in the inferior leads (blue arrows) are observed, which strongly suggest acute myocardial infarction. The baseline rhythm of the patient was long-standing persistent atrial fibrillation with nonselective His-bundle pacing. (B) Changes in electrocardiographic findings from before to after hospitalization. ST-segment elevation at the time of arrival gradually decreased, along with the presence of negative T-wave inversion after percutaneous coronary intervention. At 6 months, the ST-T changes had recovered mostly to normal, with preserved R-wave amplitudes in leads V_2_ to V_4_.

Diagnosis of ST-segment changes is challenging in patients with left bundle branch block. Although several criteria for ECG have been established to diagnose myocardial infarction in intrinsic left bundle branch block, their low variable sensitivity (18%-81%) was insufficient when applied to the paced rhythm of right ventricular pacing.^1^^,^^2^ Furthermore, the drifting horizontal line on ECG, due to chest pacing and the f-wave of atrial fibrillation, especially in this case, could make it difficult to measure the minimal difference in amplitude along with the criteria. A temporary inhibition of ventricular pacing to expose underlying intrinsic rhythm is not typically acceptable, because the “T-wave cardiac memory” may affect electrocardiographic changes generated by pacing rhythm and emerging myocardial injury.^3^ In contrast, HBP has emerged as the first physiological pacing using an intrinsic conduction system, and most ventricular activation is the same as native normal ventricular activation, except for a small part of the basal ventricular septum in nonselective HBP. Thus, concordant electrical dynamics between the QRS complex and T-wave on ECG can reveal a feature similar to the native QRS and ST-T changes during nonselective HBP and can help diagnose ischemic abnormalities accordingly.

Early diagnosis of acute myocardial infarction is essential for prompt coronary reperfusion, which is associated with a favorable prognosis and reduced malignant complications. In this case, marked ST-segment elevation on ECG made it possible for young fellows to be aware of the typical key signs of acute myocardial infarction, resulting in a shorter door-to-balloon time of 80 minutes (optimal timing, <90 minutes) even on a Sunday morning, with a favorable clinical course and normalization of the electrocardiographic change after 6 months.

## Funding Support and Author Disclosures

Drs Yanagisawa and Shibata are affiliated with a department sponsored by Medtronic Japan. All other authors have reported that they have no relationships relevant to the contents of this paper to disclose.
