# Supplementary material for: Acute Chest Pain and ST-Segment Changes in a Patient After Permanent His-Bundle Pacing Pacemaker Implantation
Source: JACC Case Rep. 2025 Feb 12;30(8):103203. doi: 10.1016/j.jaccas.2024.103203 (PMC12046825; doi:10.1016/j.jaccas.2024.103203)
Supplement: Supplemental Material [file mmc1.docx]

**Supplemental Figure 1. Coronary angiography and intervention**

**
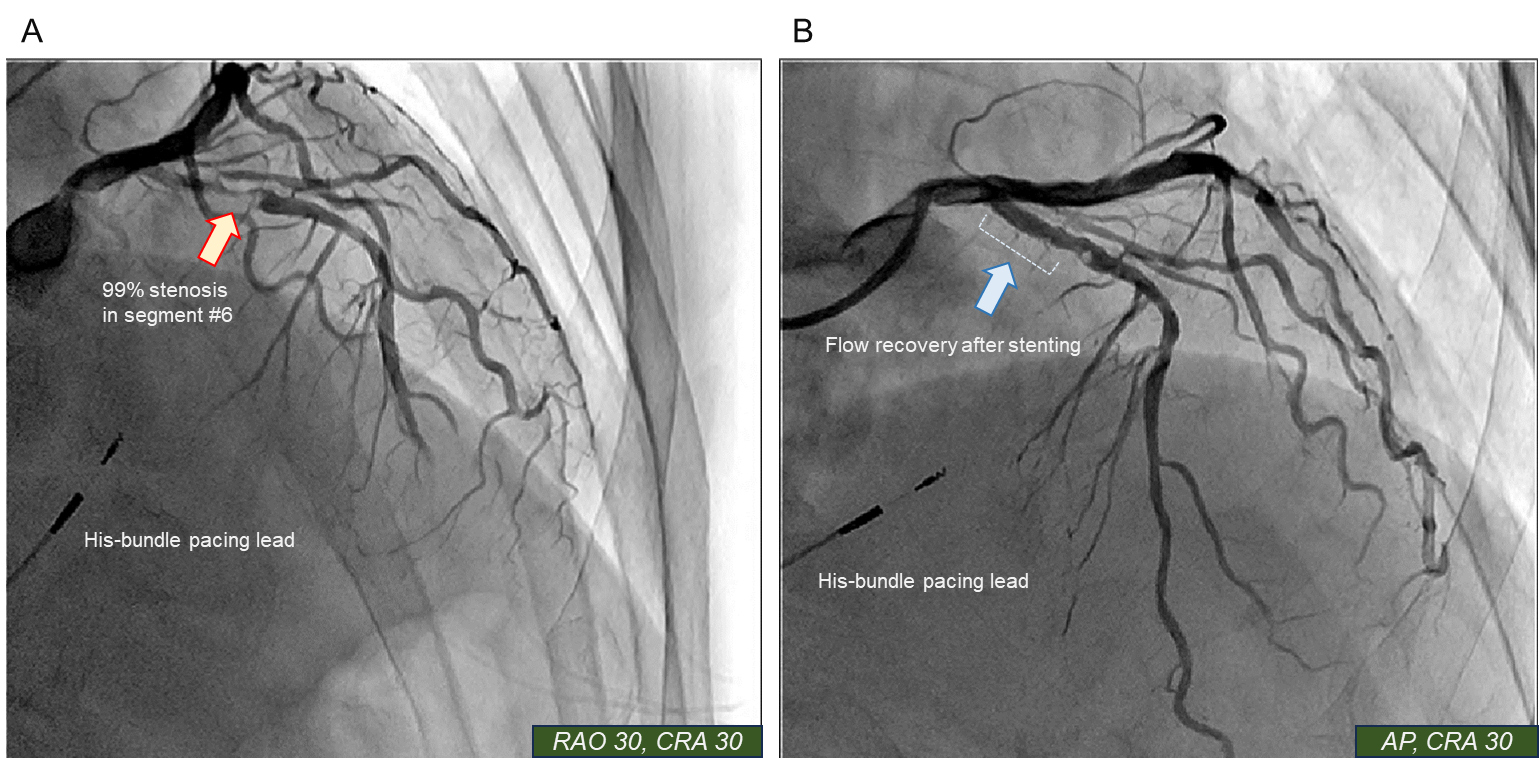
**

(A) A 99% stenosis of the proximal lesion in the left anterior descending artery is observed.

(B) Drug-eluting stent implantation of SYNERGYTM XD following thrombus aspiration of the target lesion recovers the coronary flow. Note that the door-to-balloon time and procedural starting time to reperfusion time in this case are favorable at 80 and 30 min, respectively.

CRA, cranial view; RAO, right anterior oblique view

**Supplemental Figure 2. Trends of the pacing threshold and lead impedance of the pacemaker**

**
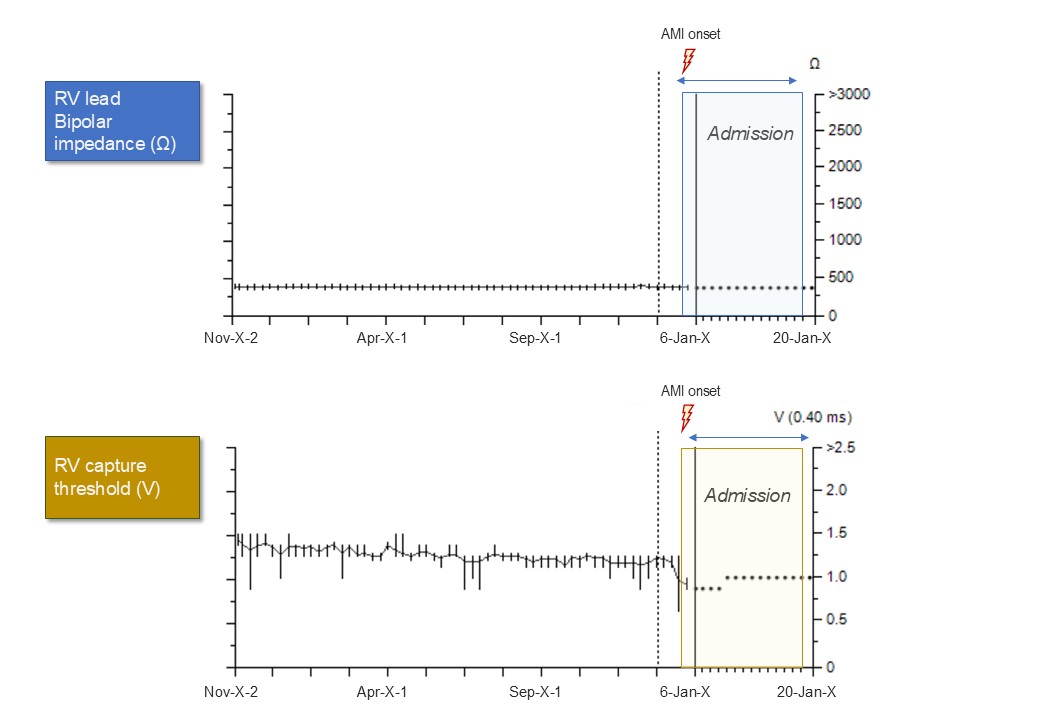
**

No remarkable change in the pacing threshold or lead impedance is observed both before and after hospitalization. A face-to-face interrogation result one month before the hospitalization reveals pacing thresholds of 0.75 V and 1.0 V at 1.0 ms (1.0 V and 1.5 V at 0.4 ms) for His-bundle capture and right ventricular capture, respectively, which are similar to the His-bundle and right ventricular capture thresholds of 0.75 V and 0.75 V at 1.0 ms (1.0 V and 1.25 V at 0.4 ms), respectively, 2 months after the discharge. There is no sensing of the intrinsic R-wave behind the pacing rhythm. The differential diagnosis for the type of His-bundle capture between selective and non-selective pacing on electrocardiography was based on the presence of an isoelectric interval in all leads, pseudo-delta wave, and change in R-wave amplitude during the threshold test at the follow-up period.^1^ Furthermore, the absence of plateaus, notching, and/or slurring in leads I, V1, and V4–6, as well as V6 R-wave peak time < 100 ms, are indicative of non-selective His-bundle pacing, which can distinguish it from myocardial capture.^2^ In this case, electrocardiography before the admission (Figure 1B), which showed no notches/slurs in leads I, V1, and V4–6; a V6 R-wave peak time of 80 ms; and a paced QRS duration of 112 ms, confirmed the non-selective His-bundle pacing according to standard classification.^2,^^3^

**References**

1. Burri H, Jastrzebski M, Vijayaraman P. Electrocardiographic Analysis for His Bundle Pacing at Implantation and Follow-Up. *JACC Clin Electrophysiol* 2020;6:883-900.

2. Jastrzębski M, Moskal P, Curila K, et al. Electrocardiographic characterization of non-selective His-bundle pacing: validation of novel diagnostic criteria. *Europace* 2019;21:1857-64.

3. Burri H, Jastrzebski M, Cano Ó, et al. EHRA clinical consensus statement on conduction system pacing implantation: endorsed by the Asia Pacific Heart Rhythm Society (APHRS), Canadian Heart Rhythm Society (CHRS), and Latin American Heart Rhythm Society (LAHRS). *Europace* 2023;25:1208-36.
